# Hemophagocytic Lymphohistiocytosis Following Skin and Soft Tissue Infection in a Patient With Human Immunodeficiency Virus

**DOI:** 10.7759/cureus.15702

**Published:** 2021-06-16

**Authors:** Omid Yazdanpanah, Lea M Monday, Asra N Shaik, Zachary Cantor, Jie Chi

**Affiliations:** 1 Internal Medicine, Wayne State University School of Medicine, Detroit, USA; 2 Internal Medicine, John D. Dingell VA Medical Center, Detroit, USA

**Keywords:** hemophagocytic lymphohistiocytosis (hlh), skin and soft tissue infection, human immunodeficiency virus (hiv) infection, “pancytopenia”, clinical hematology

## Abstract

Hemophagocytic lymphohistiocytosis (HLH) is a systemic inflammatory syndrome of inappropriate immune cell activation which can be rapidly fatal if not recognized and treated. Here we discuss a case of a 26-year-old male with HIV on antiretroviral therapy who presented with sepsis secondary to soft tissue infection and ultimately progressed to multi-organ dysfunction despite broad-spectrum antibiotics and an improvement in soft tissue infection. Continued fever and pancytopenia without an explanation found during additional infectious and rheumatologic testing eventually led to bone marrow biopsy and laboratory criteria consistent with HLH. Although pancytopenia is a common finding in patients with HIV, here it marked a more rapidly progressing and fatal disease, HLH. Here we highlight the difficulty in identifying and diagnosing this rare condition, including a discussion of the characteristics, outcomes, underlying etiologies, and treatment of HLH in patients with HIV.

## Introduction

Hemophagocytic lymphohistiocytosis (HLH) is a rare and life-threatening syndrome characterized by the overproduction of inflammatory cytokines and hemophagocytosis, leading to multi-organ failure [1,2]. The HLH has a high mortality rate of 50-70%, especially in immunocompromised individuals [2]. Diagnosis of HLH can be particularly challenging in immunocompromised patients (such as those with HIV infection) due to concomitant hematologic abnormalities.

Here we present a case of HLH in a 26-year-old patient with HIV initially presenting with sepsis secondary to soft tissue infection that ultimately progressed to multiorgan dysfunction. Systematic workup to clinch the final diagnosis was muddled by several confounders, ultimately delaying diagnosis and treatment for HLH in this patient. Despite appropriate immunosuppressive management and initial clinical improvement, the disease course progressed, and the patient expired due to complications of the disease. Through this case, we examine the steps critical for early diagnosis and management of HLH. In addition, we discuss the benefits of early immunosuppressive treatment, the risks of secondary opportunistic infections, and ways in which treatment delays can lead to irreversible multi-organ failure.

## Case presentation

A 26-year-old gentleman with a past medical history of HIV and schizoaffective disorder presented with left arm pain after a physical assault. He had been on antiretroviral therapy for one year with CD4 count 227 (500-1200 cells/m3) and an undetectable viral load. He had no recent sex partners in the last six months, no pets, and no recent travel. History was significant for a recent physical assault 16 days before this admission, resulting in a left forearm skin abscess. He underwent surgical irrigation and debridement of left forearm abscess and completed a two-week course of antibiotic treatment, initially intravenous vancomycin followed by oral trimethoprim/sulfamethoxazole. Then was transferred to a rehabilitation facility. 

On the first day following discharge, the patient became septic again and was readmitted to the hospital. Initial vital signs on admission revealed hypotension with blood pressure 85/45 mmHg (90/60-120/80 mmHg), tachycardia with heart rate 123 bpm (60-100 bpm), and fever with temperature 39.1 C (36.5-37.2 C). Oxygen saturation was normal. Physical exam showed erythema around the surgical wound without purulence or fluctuance to suggest an abscess on the left forearm. A plain radiogram of the left forearm showed no evidence of fracture, Computed tomography (CT) was only suggestive of hematoma. Initial laboratory tests revealed a pancytopenia with hemoglobin 6.7 g/dL (13.5-17.5 g/dL), white blood cells 2.0/mm3 (4.5-11.0/mm3), and platelets 74 (150-400/mm3). Creatinine on admission was within the normal range, 1.05 mg/dL (0.6-1.2mg/dL).

The patient was initially started on cefepime and vancomycin for presumed sepsis secondary to the infected surgical wound on the left forearm. Despite the initiation of broad-spectrum antibiotics, he continued to experience spike high fevers >39C and developed septic shock requiring intensive care unit admission. Bacterial and fungal cultures from the blood and surgical wound were negative. COVID-19 polymerase chain reaction (PCR) was negative. Viral quantitative PCR testing for Epstein-Barr virus (EBV) and cytomegalovirus (CMV) were negative, as was nasopharyngeal swab PCR for respiratory viruses including influenza, parainfluenza, and human metapneumovirus. A CT abdomen revealed mild hepatosplenomegaly. Rheumatological studies including anti-double-stranded DNA, antinuclear antibodies (ANA), antineutrophilic cytoplasmic antibody (ANCA), and extranuclear antibodies (Ro, Sm, RNP, La, Scl-70, Jo) were negative. Complement levels were reduced, C4 was 9 mg/dl (16-48mg/dl) and C3 was 46 mg/dl (80-160mg/dl).

The patient’s pancytopenia continued to worsen, and he was transfused multiple units of red blood cells. Given the continued pancytopenia of unidentified cause, bone marrow biopsy was performed. It revealed 80% cellular marrow with myeloid hyperplasia, plasmacytosis, dyserythropoiesis and erythrophagocytosis. Laboratory data at that time showed hemoglobin 6.7 g/L (13.5-17.5 g/dL), absolute neutrophil count 0.8 (3-5%), platelets 34 (150-400k/mm3), creatinine 2.1 mg/dL (0.6-1.2mg/dL), LDH 172 u/L (<150mg/dL), triglycerides 243 mg/dL (35-160mg/dL), D-dimer 23.43mg/L (<0.46mg/L), ferritin 8355 mg/dL (15-200 ng/mL), interleukin 2 receptor (CD25) 4370pg/mL (<2500pg/mL), and fibrinogen 337. Given this constellation of clinical symptoms, laboratory abnormalities, and bone marrow findings, the patient was diagnosed with HLH based on the HLH-2004 diagnostic criteria as demonstrated in Table 1 [3].

**Table 1:** HLH Diagnostic criteria according to HLH-2004 study and the criteria fulfilled by this patient: Five or more fulfilled criteria are sufficient to diagnose HLH [3]. ᵅ Peripheral blood cytopenia, with at least two of the following: hemoglobin <9 g/dL; platelets <100,000/microL; absolute neutrophil count <1000/microL ᵇ Hypertriglyceridemia (fasting triglycerides >265 mg/dL) and/or hypofibrinogenemia (fibrinogen <150 mg/dL) ᵈ Hemophagocytosis in bone marrow, spleen, lymph node, or liver ᵉ NK cell activity <10 lytic units ᵍ Elevated soluble CD25 (soluble IL-2 receptor alpha) two standard deviations above age-adjusted laboratory-specific norms

Diagnostic Criteria	HLH-2004 Study Fulfillment %	Our Patient
Fever ≥ 38.5°C	95%	X
Splenomegaly	89%	X
Bicytopenia ᵅ	92%	X
Hypertriglyceridemia and/or hypofibrinogenemia ᵇ	90%	
Hemophagocytosis ᵈ	82%	X
Low or absent NK cell activity ᵉ	71%	
Ferritin >500 ng/mL	94%	X
Elevated soluble CD25 ᵍ	97%	X

On day 17 of admission, HLH treatment was initiated with etoposide and dexamethasone as outlined in the HLH-2004 protocol. Cyclosporine had ultimately deemed a poor therapeutic option due to multiple comorbidities. The patient was severely cachectic and required enteral nutrition via tube feeds. His course was further complicated by hospital-acquired infections including *Clostridioides difficile* diarrhea and central line-associated bloodstream infection. Although the patient initially showed some clinical improvement after immunosuppression with declining ferritin, his pancytopenia subsequently worsened and he succumbed to his disease two months after he was admitted.

## Discussion

HLH is a life-threatening systemic inflammatory condition marked by the inappropriate activation of the mononuclear phagocytic system resulting in excessive cytokine production and multi-system organ failure [1,2]. HLH can be classified as either primary (familial) or secondary (acquired) depending on the underlying pathophysiology [1]. In primary HLH, inherited genetic defects result in malfunction of proteins required for normal cytotoxic functioning of T- lymphocytes and natural killer (NK) cells. Patients with primary HLH typically present by age two, although rare forms in adolescents and adults have been reported [4]. Aggressive combined immunosuppression and chemotherapy followed by allogeneic bone marrow transplant are required to prevent recurrence or malignant transformation [1,2]. In contrast, secondary (acquired) HLH occurs due to defects in lymphocyte and NK cell function caused by an underlying infectious, autoimmune, or malignant disorder that triggers activation of the monocyte phagocytic system [2]. 

Clinical characteristics and outcomes of adults diagnosed with acquired HLH have been explored in three large case series [5-7]. Otrock and Eby described 73 adults diagnosed with HLH over an 11-year period in the United States (US). Median age at diagnosis was 51 years with a 30-day mortality of 27.4%. The etiology of HLH was due to infection (41%) or malignancy (29%) in most cases [5]. A second large case series published around the same time by Parikh and colleagues described 62 HLH patients diagnosed over 16 years. Median age at HLH diagnosis was 49 years, most commonly triggered by malignancy (52%) or infection (34%) and with a 30-day mortality of 44% [6]. Lastly, a large multi-center French study described 162 HLH patients diagnosed at a median age of 48 years. Thirty-day mortality was 20% and again the majority of HLH cases were precipitated by malignancy (57%) or infection (25%) [7]. All three series noted that diagnosis was often delayed due to the nonspecific clinical findings and overlap in symptoms and laboratory abnormalities with the illness triggers themselves including infection and malignancy [5-7]. HLH manifests clinically as a febrile illness with multi-system involvement. Patients may present with fever and be found to have abnormalities including organomegaly (most commonly splenomegaly), cytopenia (in one or more hematopoietic cell lines), hemophagocytosis on bone marrow aspirate, and laboratory elevations in triglycerides, ferritin, and lactate dehydrogenase (LDH) [2,4-7]. Several other conditions can present similarly including infections and sepsis, hematologic disorders and malignancies, drug reactions, and fevers of unknown origin [8,9]. Clinicians must prioritize an assessment of organ involvement, including bone marrow and liver dysfunction while monitoring for immune hyperactivation since HLH requires prompt treatment once the diagnosis is established or strongly suspected.

HLH in patients with HIV infection is well-reported and often has a poor prognosis [10,11]. A recent 2020 systemic review described 52 cases of concomitant HIV and HLH diagnosed at a median age of 38 years with an overall mortality of 40% [10]. The median CD4 count was low (41 cells/m3) and only 7 of 52 patients were virally suppressed [10]. The underlying etiologies of acquired HLH in HIV patients include concomitant infection and malignancy similar to non-HIV patients. However, uncontrolled replication of the HIV virus itself can also cause HLH [10-12]. A series of patients with HLH acquired due to infection found concomitant HIV was present in 50% of the patients, although it was the additional co-infections rather than HIV itself that was thought to be the trigger for HLH in most cases [12]. Many clinical symptoms are present in both HLH and advanced HIV including fever, splenomegaly, pancytopenia, and hyperferritinemia [13]. While not pathognomonic, phagocytosis of nucleated cells is strongly correlated with the diagnosis of HLH [7,14]. However, in a case series of autopsies, 11 of 54 AIDS patients had confirmed hemophagocytosis, further demonstrating the difficulty in making a clear HLH diagnosis in these patients [15]. Overall, the propensity for underlying infection and malignancy combined with the overlap in clinical manifestations, laboratory abnormalities, and bone marrow findings of HLH and advanced HIV disease requires a high index of suspicion to make this diagnosis in a timely manner.

In the case of our patient, the diagnosis of HLH was delayed by the investigation of sepsis in the setting of HIV and recent skin and soft tissue infection. Eventually, it was the persistent pancytopenia combined with abnormalities in triglycerides, ferritin, and soluble CD25 which prompted a bone marrow biopsy and ultimately the diagnosis of HLH based on HLH-04 criteria [3]. Given his CD4 count of above 200 cells/m3 on admission, it is unlikely that uncontrolled HIV itself led to HLH, but rather the presence of underlying soft tissue infection which had been present for some time prior to admission. The specific underlying infections most reported to cause HLH in HIV patients include histoplasmosis and other fungi, pneumocystis, mycobacteria, and viruses such as EBV and CMV; however, underlying bacterial infections are rarely reported as a cause [10]. The negative viral serology and lack of respiratory, skin, and gastrointestinal abnormalities make these other infections unlikely.

Treatment of HLH is well established in pediatric patients based on Histiocyte Society provided guidelines for HLH diagnosis and management including the HLH-2004 treatment protocols [3]. Protocols often contain etoposide, dexamethasone, and cyclosporine A which have strong activity against cytotoxic T/natural killer cells (NK-cells) and macrophage proliferation. HLH in adults is a more heterogeneous syndrome that requires an individualized approach depending on the underlying trigger, genetic background, and disease course and severity. Data regarding the generalizability of these diagnostic and treatment criteria to adult patients, and especially those with HIV, are largely lacking [3,10].

## Conclusions

Here we presented a case of HLH in an HIV patient that was possibly triggered by an underlying bacterial soft tissue infection. In HIV patients with febrile pancytopenia, the differential is wide and suspicion for HLH should be maintained if treatment of an initial underlying infection does not result in clinical improvement. HIV and HLH overlap in clinical manifestations, laboratory abnormalities, and bone marrow findings. A high index of suspicion is required to make diagnose HLH in HIV patients in a timely manner. HLH treatment in adults is heterogeneous, based on expert opinion, and often determined on a case-by-case assessment of the underlying cause and comorbidities. 
